# Existence of Molten Globule State in Homocysteine-Induced Protein Covalent Modifications

**DOI:** 10.1371/journal.pone.0113566

**Published:** 2014-11-18

**Authors:** Tarun Kumar, Gurumayum Suraj Sharma, Laishram Rajendrakumar Singh

**Affiliations:** Dr. B. R. Ambedkar Center for Biomedical Research, University of Delhi, Delhi, India; Aligarh Muslim University, India

## Abstract

Homocysteine thiolactone is a toxic metabolite produced from homocysteine by amino-acyl t-RNA synthetase in error editing reaction. The basic cause of toxicity of homocysteine thiolactone is believed to be due to the adduct formation with lysine residues (known as protein N-homocysteinylation) leading to protein aggregation and loss of enzyme function. There was no data available until now that showed the effect of homocysteine thiolactone on the native state structural changes that led to aggregate formation. In the present study we have investigated the time dependent structural changes due to homocysteine thiolactone induced modifications on three different proteins having different physico-chemical properties (cytochrome-c, lysozyme and alpha lactalbumin). We discovered that N-homocysteinylation leads to the formation of molten globule state—an important protein folding intermediate in the protein folding pathway. We also found that the formation of the molten globule state might be responsible for the appearance of aggregate formation. The study indicates the importance of protein folding intermediate state in eliciting the homocysteine thiolactone toxicity.

## Introduction

Homocysteine is a sulfur containing toxic metabolite produced as a byproduct in methionine metabolism pathway. This toxic homocysteine is known to metabolize to methionine by remethylation or to cystiene by trans-sulfurylation [1]. However, mutations in the homocysteine metabolizing enzymes, cystathionine β-synthase (CBS) or methylene tetrahydrofolate reductase (MTHFR) cause an impaired ability to metabolize the toxic homocysteine resulting in an increased levels of cellular and plasma homocysteine [2], [3]. The increased accumulation of plasma homocysteine results in homocystinuria and the symptoms include arteriosclerosis, osteoporosis, mental retardation, thrombosis, dislocated eye lenses and neurodegenerative pathologies such as dementia, Parkinson's and Alzheimer's diseases [4], [5], [6], [7]. The serum level of homocysteine in healthy adults is 5–10 µM, it may range from 15–20 µM in mild form but in case of hyperhomocysteinimia it may rise up to 500 µM [8], [9]. The toxic effect of homocysteine has been believed due to the formation of homocysteine thiolactone (HTL), which is synthesized from homocysteine by amino acyl t-RNA synthetase in proofreading or error editing reaction mechanism whenever homocysteine gets incorporated in place of methionine by mistake during translation process [10], [11], [12], [13]. It has been demonstrated that HTL preferentially forms amide bonds with *ε*-amino group of lysine residues of protein in a non-enzymatic mechanism; a process referred to as “protein N-homocysteinylation” [13] which results in various kinds of toxic effects on macromolecules including loss of enzymatic activity, generation of oxidative stress within the cell and autoimmune response generation against N-homocysteinylated self proteins [14].

It has been known that protein N-homocysteinylation results in the formation of toxic multimers, aggregate or amyloid and therefore, this has been considered to be a risk factor for neurodegeneration [15], [16], [17], [18], [19], [20], [21]. Various studies have also shown that the protein aggregation can also be induced *in vitro* by partially folded; molten globule (MG) states [22], [23]. Molten globule states are indeed compact denatured protein folding intermediates that exist between the native and denatured states [24]. MG state has attracted much attention in recent years because it is believed to be involved in many biological processes [25], [26], [27], [28], [29], [30]. It has the following common structural characteristics: (i) presence of intact secondary structure, (ii) loss of tertiary interactions, (iii) presence of loosely packed hydrophobic core that increases the hydrophobic surface accessibility to solvent environment and (iv) 10–30% increase in the radius of gyration [29], [31], [32], [33]. Studies on covalent modification of native proteins by glycating agents that target lysine residues (similar to HTL induced covalent modifications) have also been shown to result in the formation of molten globule states ultimately leading to aggregate formation [23]. Since covalent modification by HTL has been known to induce aggregate/amyloid formation, it is important to investigate if HTL induced covalent modification of proteins also results in formation of MG states for which the detailed structural consequences that HTL modification has on the native state of proteins have to be understood.

Most studies reported earlier on the structural consequences of homocysteine on proteins were however made at time intervals ranging from 12–72 hours [15], [17], [19], [34], [35], [36] where proteins have already formed aggregate. To the best of our knowledge there are no reports of the effect of protein N-homocysteinylation on the structural characteristics of native proteins at different time intervals. Therefore, what effects HTL exposure results in the native state conformation of the protein has not been properly understood. Here, we investigated the effect of protein N-homocysteinylation on the conformation of three different proteins having different physico-chemical properties and different lysine contents (cytochrome-c from bovine heart, bovine alpha lactalbumin and lysozyme from chicken egg white) at different time intervals. The present work clearly demonstrates that protein N-homocysteinylation results in the formation of molten globule state- an important protein folding intermediate in case of cytochrome-c (cyt-c) and alpha lactalbumin (α-LA) while there exists no structural changes on lysozyme. We also found that molten globule state might be responsible for the formation of aggregate due to protein N-homocysteinylation. Our results indicate the importance of the protein folding intermediate in eliciting the toxic effect of protein N-homocysteinylation.

## Materials and Methods

### Materials

Cytochrome c (from bovine heart), lysozyme (from chicken egg white), alpha lactalbumin (from bovine milk), DL-homocysteine thiolactone hydrochloride (HTL) and 8-Anilinonaphthalene-1-sulphonic acid were purchased from Sigma-Aldrich chemical Co. Potassium chloride, di-potassium hydrogen phosphate and potassium di-hydrogen phosphate were purchased from Merck, India. Dithiobis(2-nitrobenzoic acid), the Ellman's reagent was also purchased from Sigma-Aldrich chemical Co. Guanidium hydrochloride (Gdmcl) was purchased from M.P. Biomedicals. Unless otherwise stated, all chemicals were used without further purification and double distilled water was used as aqueous phase.

### Analytical Procedures

Lysozyme, cyt-c and α-LA solutions were dialyzed extensively against 0.1 M KCl at pH 7.0 at ∼4°C. Protein stock solutions were filtered using 0.22-µm Millipore syringe driven filter. Cytochrome c was oxidized using 0.01% potassium ferrocyanide before dialysis. All the proteins gave a single band during polyacrylamide gel electrophoresis (see Figure S1). Concentration of the protein solutions was determined experimentally using the molar absorption coefficient (*ε*) values 3.9×10^4^ M^−1^ cm^−1^ at 280 nm for Lysozyme [37], 1.06×10^5^ M^−1^ cm^−1^ at 409 nm for cyt c [38] and 29210 M^−1^ cm^−1^ at 280 nm for α-LA [39]. All solutions for optical measurements were prepared in the desired degassed buffer (0.05 M phosphate buffer, pH 7.0). Since pH of the protein solution may change on the addition of HTL, pH of each solution was also measured after addition of HTL. We observed no significant change in the pH of the protein solutions after addition of HTL.

### Modification of proteins (cyt c, α-LA and lysozyme) by HTL and sulfhydryl estimation using Ellman's reagent

Proteins were treated with different concentrations of HTL (1 mM, 2 mM, 5 mM and 10 mM) and then incubated (18 hours for cyt-c, 4 hours for α-LA and 24 hours for lysozyme) at 25°C in 0.05 M phosphate buffer of pH 7.0. Aliquotes of proteins modified with HTL were first precipitated down with 10% TCA to remove unbound HTL. Protein pellets were collected and then resolubilized in phosphate buffer, pH 7.0. The levels of thiol groups in control and homocysteinylated protein samples were assayed using Dithiobis(2-nitrobenzoic acid), the Ellman's reagent. The absorbance of the samples were measured at 412 nm, using a 1 cm path-length cuvette. The amount of 5′-nitrothiobenzoate released was calculated from the molar extinction coefficient of 13,700 M^−1^ cm^−1^ [40], [41], [42].

### Dynamic light scattering (DLS) measurements

Size distribution of the particles present in the protein sample were obtained using a Zetasizer Micro V/ZMV 2000 (Malvern, UK). Measurements were made at a fixed angle of 90° using an incident laser beam of 689 nm. Fifteen measurements were made with an acquisition time of 30 seconds for each sample at sensitivity of 10%. The data was analysed using Zetasizer software provided by the manufacturer to get hydrodynamic diameters, and polydispersity which is a measure of the standard deviation of the size of the particles. The protein concentration was 2.0 mg/ml. All measurements were performed at 25°C.

### Circular Dichroism (CD) Measurements

CD measurements were made in a Jasco J-810 spectropolarimeter equipped with peltier controller at 25°C with six accumulations. Protein concentration used for the CD measurements was 0.5 mg/ml. Cells of 0.1 and 1.0 cm path length were used for the measurements of the far- and near-UV CD spectra respectively. Necessary blanks were subtracted. The CD instrument was routinely calibrated with D-10-camphorsulfonic acid. The molar ellipticity in units of Deg cm^2^ dmol^−1^ was determined for average residue molecular weight for the proteins used in the study.

### Fluorescence Measurements

Fluorescence spectra of the protein samples were measured in a Perkin Elmer LS 55 Spectrofluorimeter in a 3 mm quartz cell, with both excitation and emission slits set at 10 nm. Protein concentration for all the experiments was in the range of 0.06–0.07 mg/ml. For intrinsic fluorescence measurements, cyt-c, α-LA and lysozyme were excited at 295 nm, while the emission spectra were recorded from 300–500 nm. For ANS-protein binding experiments the excitation wavelength was 360 nm, and emission spectra were recorded from 400–600 nm. ANS concentration was kept 16 fold than that of protein concentration.

### Absorption spectroscopy measurements

The light scattering intensity of the protein samples were measured by monitoring the absorbance at 500 nm for cyt-c [43], 400 nm for α-LA and 450 nm for lysozyme [44] in a JASCO V-660 spectrophotometer equipped with temperature controlled cuvette holder at 25°C. The protein concentration was 1 mg/ml.

## Results

We have investigated the effect of protein N-homocysteinylation on three different proteins (cyt-c, α-LA and lysozyme) having different physico-chemical properties. The hydrophobicity indices, pI values and lysine content of these proteins are 1110, 10.5, 19 for cyt-c, 1050, 4.2, 12 for α-LA and 890, 10.7, 6 for lysozyme respectively. Concentrations of HTL for the treatment used for all the studies were 1 mM, 2 mM, 5 mM and 10 mM.

### Hydrodynamic diameter of proteins increases upon treatment with HTL

**Figure 1:**
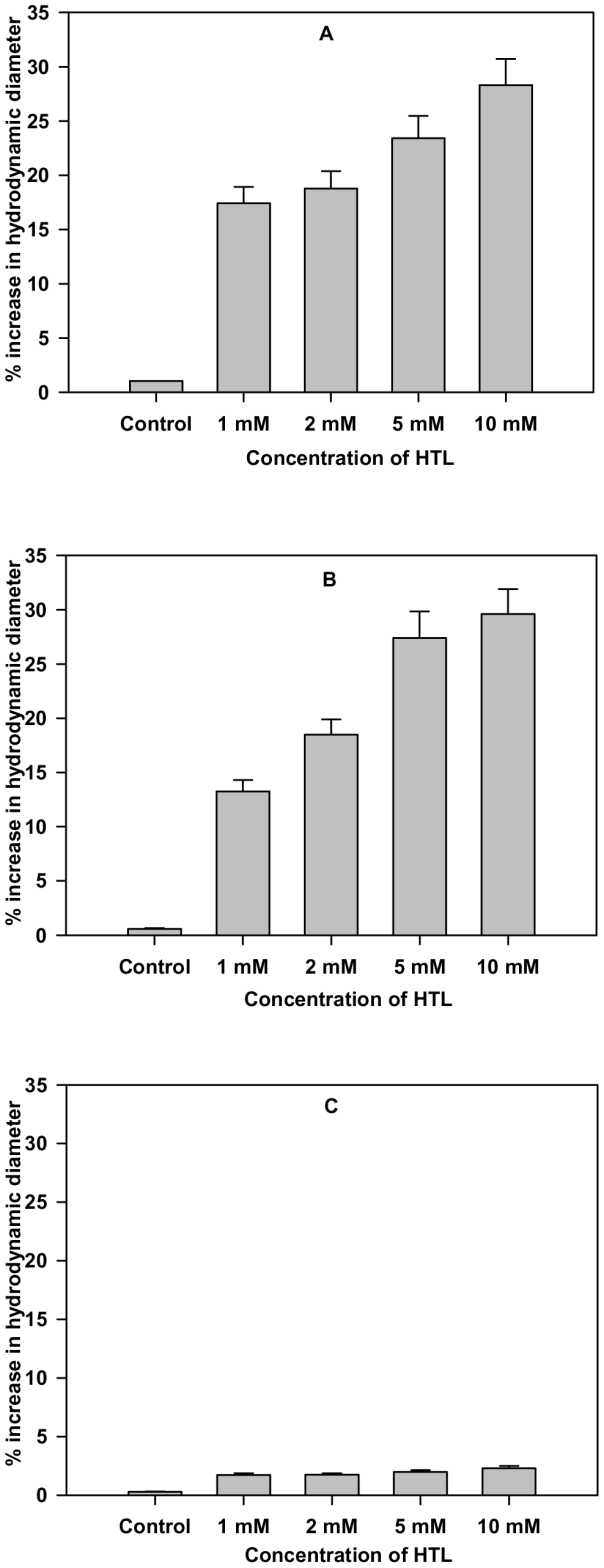
Effect of homocysteine thiolactone (HTL) on the hydrodynamic diameter of the native state of proteins. Percent increase in hydrodynamic diameter of (A) cyt-c (at 18 hours) and (B) α-LA (at 4 hours) and (C) lysozyme (at 24 hours) after modification with different concentrations of HTL.

In the present study, we have initially studied the conformational changes due to N-homocysteinylation at different concentrations of HTL using DLS as a tool at different time intervals by monitoring the hydrodynamic diameter of the proteins (Table 1). We observed significant changes in the hydrodynamic diameter of cyt-c in the time interval 4–18 hours depending on HTL concentrations and α-LA in the time interval 1–4 hours after treatment with HTL beyond which both the proteins tend to get aggregated. However, lysozyme shows no differences in the hydrodynamic diameter due to N-homocysteinylation up to the maximum time of incubation (Table 1) at all HTL concentrations used in this study. Figure 1 also shows that the changes in the maximum increase in hydrodynamic diameter of cyt-c and α-LA is around 13–30% at all HTL concentrations while in the case of lysozyme there is negligible effect on hydrodynamic diameter at all concentrations of HTL. The result suggests that hydrodynamic diameter of different proteins increases at different time intervals depending on the HTL concentration.

**Table 1:** Hydrodynamic diameter of cyt-c, α-LA and lysozyme*.

Time in hours	Hydrodynamic diameter of cyt-c (nm)				
	Native	1 mM HTL	2 mM HTL	5 mM HTL	10 mM HTL
0	2.84	2.81	2.82	2.86	2.79
1	2.81	2.83	2.81	2.84	2.78
2	2.82	2.88	2.85	2.8	2.83
3	2.75	2.8	2.85	2.85	2.84
4	2.88	2.89	2.82	2.83	2.87
6	2.81	2.79	2.72	2.85	2.98
8	2.83	2.71	2.82	2.93	3.07
10	2.78	2.78	2.88	2.98	3.28
12	2.85	2.93	2.91	3.05	3.34
14	2.83	2.95	3.03	3.24	3.41
16	2.8	3.13	3.16	3.48	3.54
18	2.87	3.3	3.35	3.53	3.58, 90.12
20	2.83	3.56	3.4	3.49, 25.26	140.8, 365.13, 831.54
22	2.91	3.45, 18.13	3.58, 20.15	116.7, 260.8, 741.8	160.56, 370.24, 840.34
24	2.88	3.52, 20.12	3.54, 24.65	120.5, 278.9, 801.4	165.45, 369.59, 845.21

Time in hours	Hydrodynamic diameter of α-LA (nm)				
	Native	1 mM HTL	2 mM HTL	5 mM HTL	10 mM HTL
0	3.42	3.55	3.46	3.54	3.58
1	3.53	3.49	3.49	3.67	3.69
2	3.52	3.56	3.58	3.69	4.14
3	3.51	3.67	3.63	4.02	4.66
4	3.44	3.98	4.1	4.51	4.64
6	3.57	4.02	4.52	6.51, 20.58	6.98, 26.47
8	3.68	6.91, 15.32	6.93, 20.13	6.88, 23.46	6.83, 30.15

Time in hours	Hydrodynamic diameter of lysozyme (nm)				
	Native	1 mM HTL	2 mM HTL	5 mM HTL	10 mM HTL
0	3.38	3.45	3.44	3.55	3.49
1	3.41	3.55	3.33	3.52	3.52
2	3.38	3.33	3.38	3.52	3.49
3	3.39	3.43	3.32	3.58	3.53
4	3.39	3.52	3.37	3.42	3.53
6	3.38	3.58	3.49	3.42	3.64
8	3.39	3.49	3.55	3.53	3.54
10	3.32	3.51	3.49	3.53	3.54
12	3.47	3.44	3.46	3.55	3.62
14	3.52	3.43	3.49	3.46	3.57
16	3.52	3.55	3.52	3.34	3.61
18	3.44	3.56	3.49	3.46	3.51
20	3.46	3.44	3.45	3.49	3.56
22	3.39	3.53	3.57	3.55	3.47
24	3.39	3.51	3.5	3.62	3.57

*Experimental error in hydrodynamic diameter measurement is in the range of 7–9%.

### Estimation of -SH groups in all the three proteins at specified time periods confirmed protein N-homocysteinylation

To verify if the changes in the conformation of the native states is due to N-homocysteinylation, we have further measured -SH content of the protein samples treated with HTL. Increase in the -SH content has been previously reported to be a signature of the protein N-homocysteinylation [5]. Table 2 shows increase in the sulfhydryl content of all the three proteins due to treatment with HTL at 4 hours for α-LA, 18 hours for cyt-c and 24 hours for lysozyme. It is seen in this table that there is an increase in the -SH groups of the proteins upon treatment with HTL at the specified time periods mentioned in the previous section. The results confirmed that each of the native protein has been N-homocysteinylated at the specified time periods.

**Table 2:** Sulfhydryl content measurement of HTL treated cyt-c, α-LA and lysozyme*.

Concentration of HTL	Sulfhydryl content (µM/mg)		
	cyt-c (At 18 hours)	α- LA (At 4 hours)	Lysozyme (At 24 hours)
0 mM	19	36	40
1 mM	753	305	391
2 mM	972	865	819
5 mM	2052	1958	1736
10 mM	2982	3098	2013

*Error in -SH content measurement is in the range of 5–8%.

### N-homocysteinylation results in the formation of molten globule state in cyt-c and α-LA but not in lysozyme

**Figure 2:**
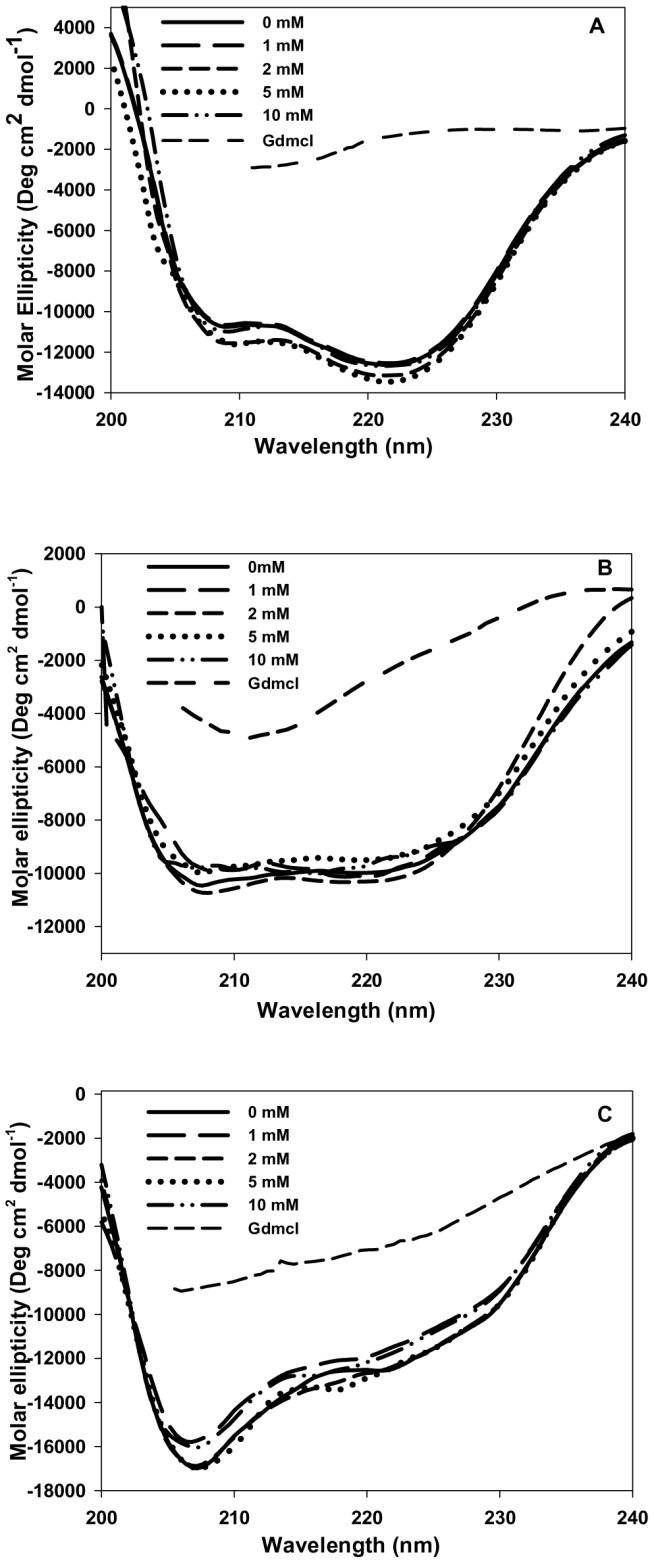
Effect of homocysteine thiolactone (HTL) on the secondary structure of the native state of proteins. Far UV CD spectra of (A) cyt-c (at 18 hours), (B) α-LA (at 4 hours) and (C) lysozyme at (24 hours) modified with different concentrations of HTL. Unfolded state control (7.0 M GdmCl) have also been shown for each protein.

**Figure 3:**
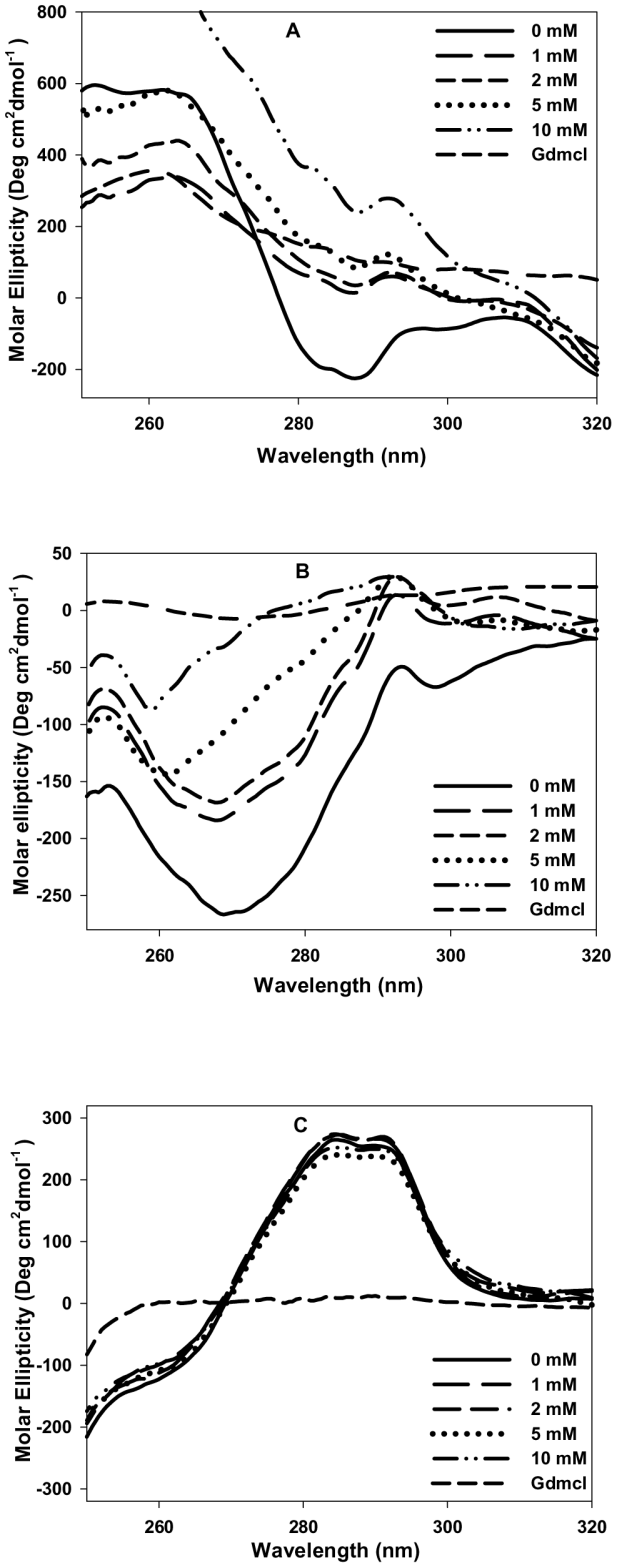
Effect of homocysteine thiolactone (HTL) on the tertiary structure of the native state of proteins. Near UV CD spectra of (A) cyt-c (at 18 hours), (B) α-LA (at 4 hours) and (C) Lysozyme (at 24 hours) modified with different concentrations of HTL. Unfolded state control (7.0 M GdmCl) have also been shown for each protein.

**Figure 4:**
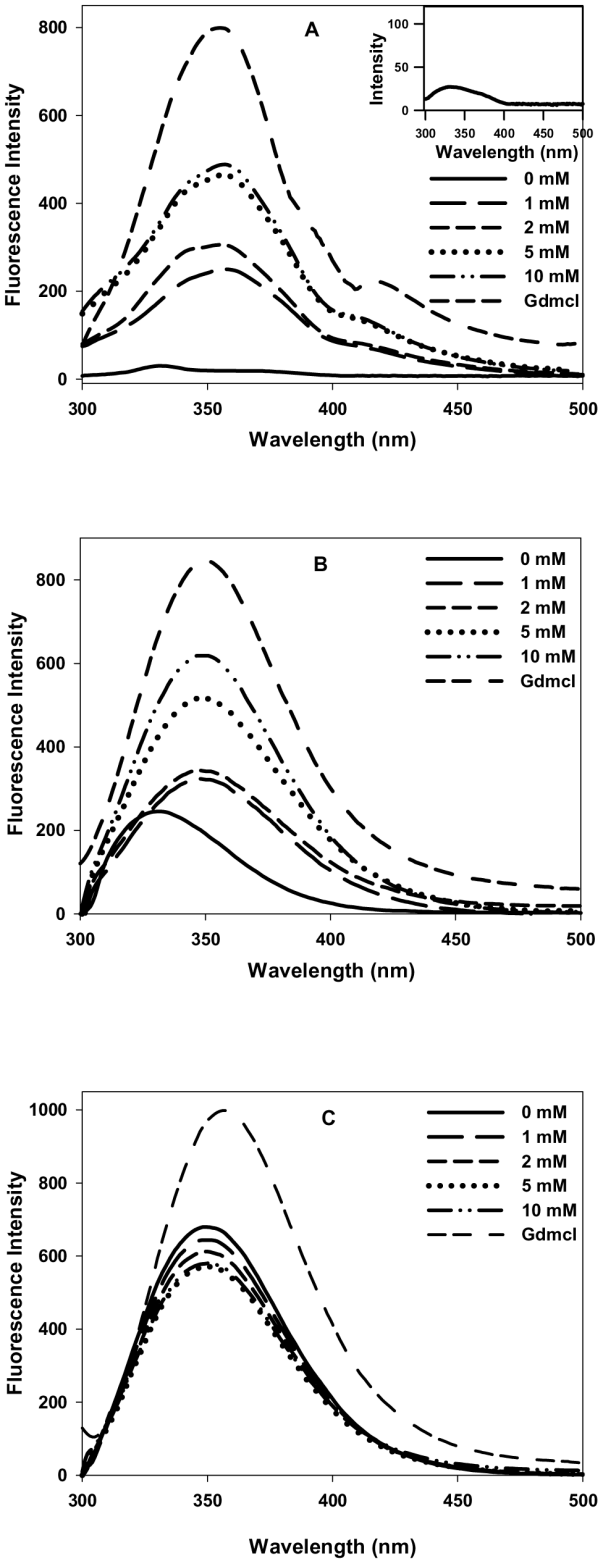
Effect of homocysteine thiolactone (HTL) on the intrinsic fluorescence of the native state of proteins. Intrinsic fluorescence spectra of (A) cyt-c (at 18 hours), (B) α-LA (at 4 hours) and (C) lysozyme (at 24 hours) modified with different concentrations of HTL. Unfolded state control (7.0 M GdmCl) have also been shown for each protein. Inset in Figure 4 (A) shows the enlarged view of the native state emission spectra of cyt-c. Excitation wavelength of 295 nm was used for all the three proteins and emissions were recorded at the wavelength range of 300–500 nm.

**Figure 5:**
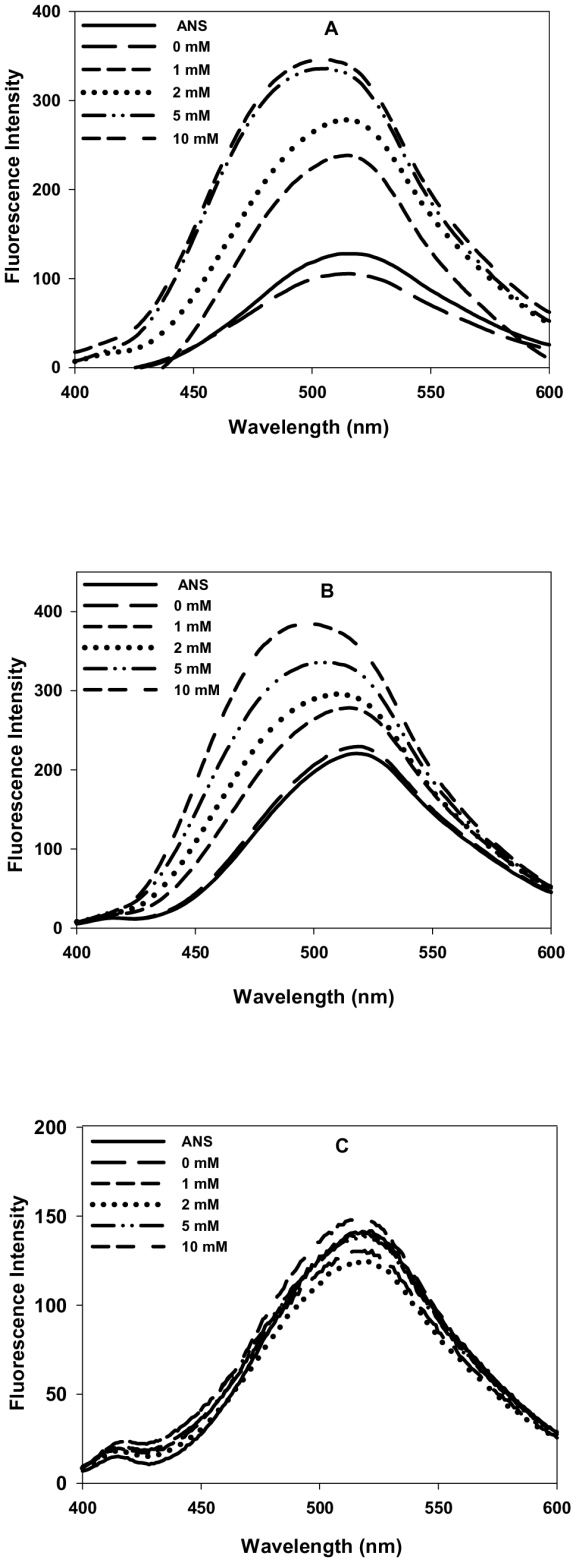
Effect of homocysteine thiolactone (HTL) on the extrinsic fluorescence of the native state of proteins. ANS binding study (A) cyt-c (at 18 hours), (B) α-LA (at 4 hours) and (C) lysozyme (at 24 hours) modified with different concentrations of HTL. Excitation wavelength of 360 nm was used and emissions were recorded at the wavelength range of 400–600 nm.

To characterize the structural changes in the proteins due to covalent modification by HTL, we measured the changes in secondary and tertiary structures of the proteins at 4 hours for α-LA, 18 hours for cyt-c and 24 hours for lysozyme by measuring the far-, near-UV CD and intrinsic fluorescence spectra of all the three HTL treated proteins. It may, however, be noted that in the case of cyt-c there appears a non-native species in the presence of 10 mM HTL at 18 hours (Table 1). We have intentionally taken 18 hours incubation time for cyt-c for structural characterization although it has one additional non-native species in our DLS study, as this time interval represents the maximum change in the native state hydrodynamic diameters of protein at all HTL concentrations and the volume fraction of the non-native species observed is negligible (volume distribution less than 1%). Figure 2 shows the secondary structural changes of the proteins treated and incubated for the respective time periods (as shown in figure legend) with different concentrations of HTL. It is seen in this figure that there is (i) neither significant alterations in the spectral properties in terms of the respective peaks of each of the protein (ii) nor the intensity (molar ellipticity) of the homocysteinylated and non-homocysteiylated proteins are changed at different concentrations of HTL. The result leads us to believe that there are no changes in the secondary structure of all the three proteins due to N-homocysteinylation. However, the near-UV CD spectra shown in Figure 3 suggests that the tertiary structures of both cyt-c and α-LA have been unfolded due to N-homocysteinylation at the specified time intervals, while the tertiary structure of lysozyme is not affected due to N-homocysteinylation at different concentrations of HTL. Figure 4 also shows an increase in the tryptophan fluorescence of cyt-c and α-LA along with very prominent red shift in emission maxima (also see Table S1) but in case of lysozyme there was no alteration in the fluorescence properties indicating that N-homocysteinylation (at the respective time periods) disrupts the tertiary interactions of the native states of both cyt-c and α-LA but does not have any perturbing effect on lysozyme. Taken together, the results indicate that N-homocysteinylation leads to different consequences on the native state structure of different proteins. Absence of tertiary structure with intact secondary structure in case of cyt-c and α-LA might indicate a molten globule state. To further verify for this possibility we have intentionally performed ANS binding experiment of these N-homocysteinylated proteins at the respective time periods (Figure 5). It is seen in Figure 5, that there was increase in the intensity of ANS fluorescence and a blue shift in case of both cyt-c and α-LA upon HTL treatment after the respective time of incubations confirming that ANS binds to both the proteins indicating the existence of molten globule state in case of cyt-c and α-LA at all HTL concentrations used in this study. No such ANS binding was observed in case of lysozyme as there was neither an apparent increase in ANS fluorescence intensity nor a blue shift. The results indicate that N-homocysteinylation at the specified time periods results in molten globule state formation in case of cyt-c and α-LA but not in lysozyme.

### N-homocysteinylation induces aggregate formation in cyt-c and α-LA but not in lysozyme at 24 hours

**Figure 6:**
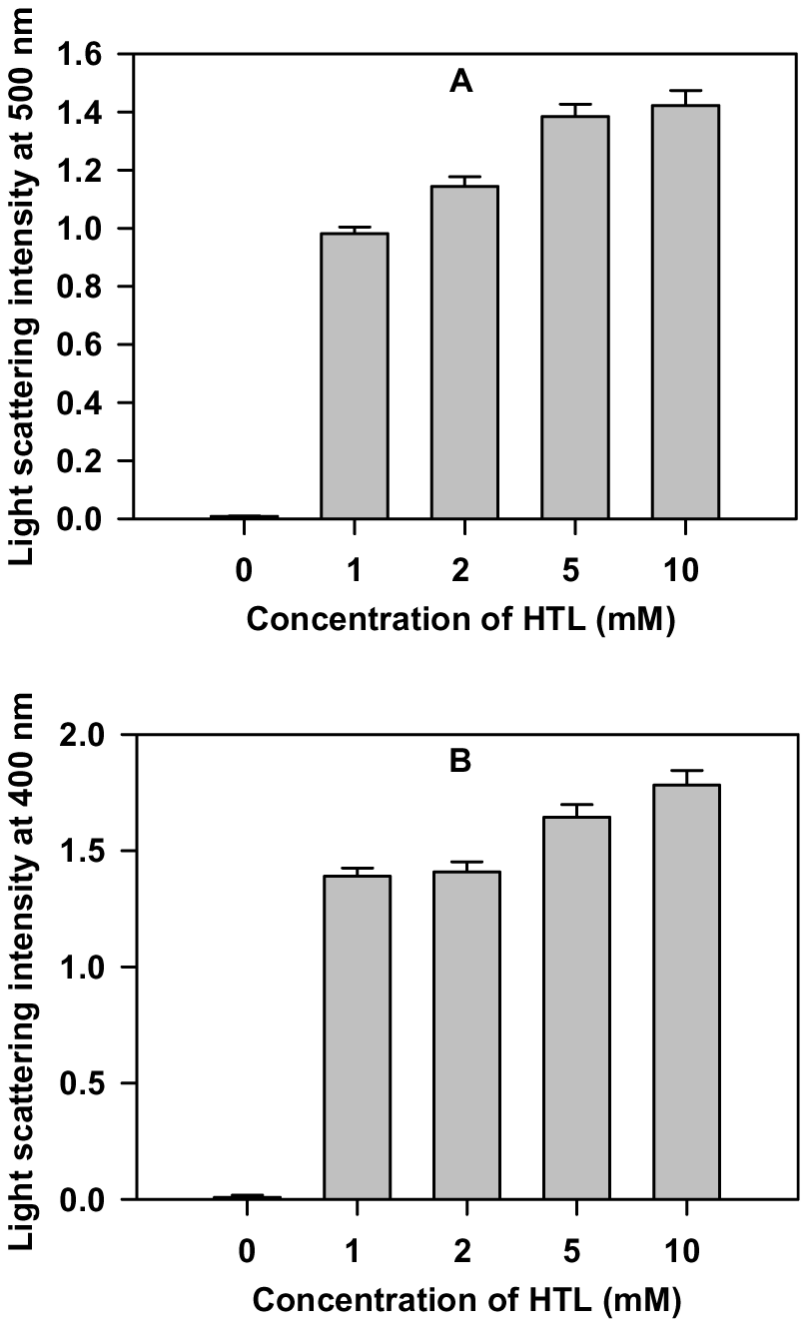
Homocysteine thiolactone (HTL) induced aggregation study of cyt-c and α-LA. Turbidometric study of (A) cyt-c (at 500 nm) and (B) α-LA (at 400 nm) modified with different concentrations of HTL at 24 hours.

In order to study the aggregation propensities of the proteins due to N-homocysteinylation, the turbidity of the modified protein samples was measured using UV-visible spectroscopy. Figure 6 shows the light scattering intensity of the proteins at the maximum time of incubation with HTL (24 hours). It is seen in this figure that both cyt-c and α-LA have been aggregated due to N-homocysteinylation. However, lysozyme was protected against HTL-induced protein aggregation (figure not shown). Since the proteins chosen in the study have different physico-chemical properties, the results indicate that protein N-homocysteinylation has different consequences on the aggregation propensities of the proteins having different physico-chemical properties.

## Discussion

To investigate for the possible effect of protein N-homocysteinylation on the native state structure of different proteins, we have first of all measured the change in the hydrodynamic diameter of the proteins at different time intervals. Results shown in Table 1 and Table 2 suggest that HTL has been incorporated to the proteins resulting in the adduct formation that affects hydrodynamic diameter of the native proteins in a time dependent manner. There is increase in the hydrodynamic diameter of the cyt-c (up to 18 hours) and α-LA (up to 4 hours). Beyond these time intervals aggregate formation starts as evidenced by large increase in the hydrodynamic diameter of the HTL modified proteins. There was no significant change in the hydrodynamic diameter of lysozyme till 24 hours. The results indicate that homocysteinylation has different consequences on the native states of different proteins. To investigate for the difference in the native state structure of the proteins due to N-homocysteinylation, we have further measured the secondary and tertiary structures of the native proteins after incubation with HTL for 4 hours in case of α-LA and 18 hours in case of cyt-c. Our results indicate that the secondary structural content (see Figure 2) for the proteins are not at all changed due to N-homocysteinylation while the tertiary structures (Figure 3 and Figure 4) have been modified differently by HTL on the different proteins. In case of lysozyme the tertiary interactions are not significantly changed while in case of cyt-c and α-LA, the tertiary interactions have been disrupted completely at the highest concentrations of the HTL used in the study. Loss of tertiary structure while having no effect on the secondary structure suggests that the native state of both the cyt-c at 18 hours and α-LA at 4 hours of incubation might be a molten globule (MG) state. Indeed MG states are characterized by the (i) presence of intact secondary structure, (ii) the loss of tertiary interactions and (iii) 10–30% increase in the radius of gyration [29], [31], [32], [33]. Interestingly, increase in the hydrodynamic diameter shown in Figure 1 (17–28% for cyt-c and 12–29% for α-LA) further evidenced that the modified proteins might exist in the MG state at the specified time periods. There is no increase in the hydrodynamic diameter of lysozyme suggesting that lysozyme does not lose its native conformation by HTL modification. If the HTL modified native state of cyt-c (at 18 hours) and α-LA (at 4 hours) is really a molten globule state then hydrophobic clusters must be exposed to the solvent due to disruption of tertiary structure. To probe for this, we have further measured the ANS binding to the proteins at the given experimental conditions as ANS specifically binds to the hydrophobic clusters exposed to the solvent [45]. Results in Figure 5 suggest that ANS has been bound to both cyt-c and α -LA but not in lysozyme. Taken together the results lead us to believe that N-homocysteinylation results in the formation of MG state in cyt-c and α -LA. It is however seen in case of lysozyme that there is no formation of such MG state since neither the tertiary structure gets unfolded nor there is increase in the hydrodynamic diameter which are prerequisite characteristic features reported for the proteins to be in molten globule state [46]. We therefore conclude that the formation of MG state due to HTL modification is dependent on the amino acid sequence of the proteins. In accordance with our results, glycating agents that target and modify proteins in the same manner by forming covalent adduct with lysine residues, also have been reported to induce MG state formation and hence aggregation [23]. At present we do not have a concrete explanation for having different effects on the native state of different proteins due to HTL induced modification. It might be possible that disruption of the tertiary structure due to N-homocysteinylation might be the limiting step to the conversion of the N-state to the MG state as the tertiary structure of lysozyme could not be disrupted due to the modification. Perhaps the incorporation of HTL at a specific lysine in each of the proteins might be responsible for opening of the tertiary structure which in the case of α-LA and cyt-c is easily accessible while is difficult to target in case of lysozyme. In support to our argument it has been shown earlier in cyt-c that incorporation of HTL in certain lysine residues does not result in significant change in the native structure while there are four lysine residues (Lys 8 or 13, Lys86 or 87, Lys 99, and Lys 100) which are susceptible to N-homocysteinylation resulting in subtle structural changes in the native structure of protein [34]. Interestingly, modification of only one single lysine (Lys 29) residue in bovine pancreatic insulin results in aggregation [15]. It has also been reported that some of the lysine residues in hemoglobin which are directly accessible to solvent environment do not get modified by HTL [35]. Based on these studies we can conclude that the modification of only selective lysine residues results in the perturbation in the structure of proteins.

It has been reported earlier that most of the end products of protein N-homocysteinylation is the formation of aggregates or amyloids [15], [16], [17], [18], [19]. We speculate that since MG states are very unstable and have exposed hydrophobic clusters, they might play an important role in the formation of aggregate or amyloid. If our speculation is true, then it is expected that both cyt-c and α -LA should form aggregate while lysozyme should not aggregate (as there are no existence of MG states in lysozyme due to N-homocysteinylation). To pursue for this possibility we have measured the aggregation propensity of the HTL adducted proteins after 24 hours by using light scattering as a tool. It is seen in Figure 6 that there is increase in the light scattering intensity in case of both cyt-c and α-LA while in case of lysozyme the scattering intensity is not at all increased (not shown in Figure). This result is also in agreement with the DLS measurement that there is existence of 2–3 aggregated species at the maximum time of incubation. Thus we conclude that the formation of MG state is responsible for different appearance of aggregates in two different proteins. One of the basic causes of homocysteine toxicity in the cells is the formation of protein aggregates or amyloids and loss of enzyme activity. The results therefore indicate the role of protein folding intermediates in eliciting the HTL toxicity. Indeed such MG state induced by HTL might be more toxic than the aggregates induced by HTL modifications. Further research should focus on these issues.

## Conclusions

In summary, we are sure of at least two things (i) N-homocysteinylation results in different consequences on the native state of different proteins: N-homocysteinylation of cyt-c and α-LA induces formation of molten globule state while there is no significant alteration in the native state structure of lysozyme. (ii) The formation of the protein aggregates due to N-homocysteinylation is protein dependent and appears to be related with the formation of molten globule state. Protein N-homocysteinylation has earlier been reported to result in different consequences on the aggregation propensities of different proteins (no aggregation to multimer and aggregate/amyloid formation) and formation of aggregates/amyloids is considered to be the basic cause of neurodegeneration [15], [16], [17], [18], [19], [20], [21]. The study therefore indicates the importance of the MG state as one of the mechanisms in eliciting homocysteine toxicity and neurodegeneration. Studies aimed at preventing the MG state formation induced by HTL modification might yield clinical implications to prevent or delay the onset of homocysteine induced proteopathies and associated disorders.

## Supporting Information

Figure S1 **SDS-PAGE profile of lysozyme (lane L1), cyt-c (lane L2) and α-LA (lane L3).** Lane M represents the protein ladder.
(TIF)

Table S1 **Fluorescence emission maxima of HTL treated cyt-c, α-LA and lysozyme.**
(DOCX)
