# Supplementary figures and images for: Existence of Molten Globule State in Homocysteine-Induced Protein Covalent Modifications
Source: PLoS One. 2014 Nov 18;9(11):e113566. doi: 10.1371/journal.pone.0113566 (PMC4236184; doi:10.1371/journal.pone.0113566)

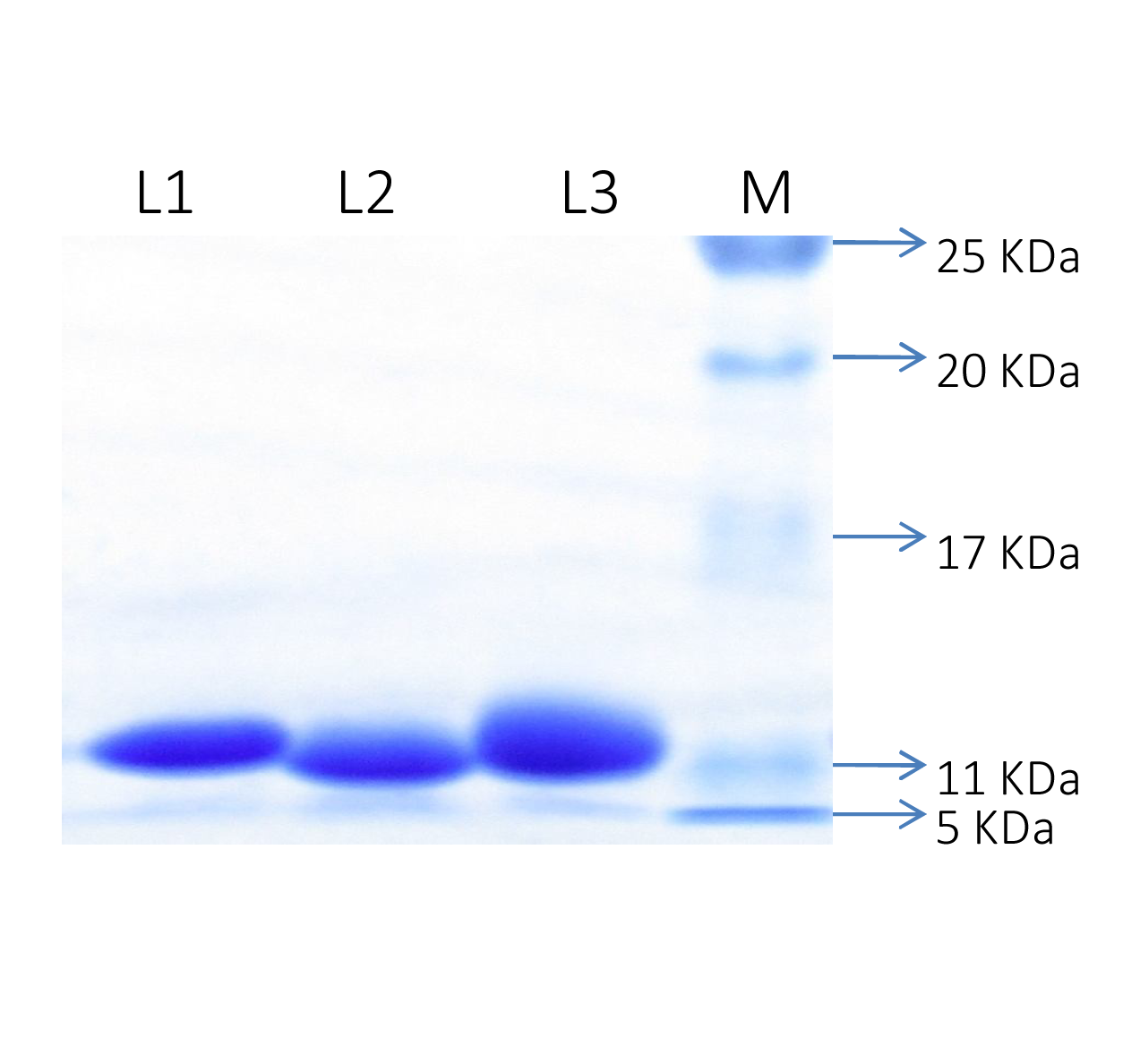

Supplement: Figure S1 — SDS-PAGE profile of lysozyme (lane L1), cyt-c (lane L2) and α-LA (lane L3). Lane M represents the protein ladder. (TIF) [file pone.0113566.s001.tif]
