# Supplementary material for: Existence of Molten Globule State in Homocysteine-Induced Protein Covalent Modifications
Source: PLoS One. 2014 Nov 18;9(11):e113566. doi: 10.1371/journal.pone.0113566 (PMC4236184; doi:10.1371/journal.pone.0113566)
Supplement: Supplementary file 2 [file pone.0113566.s002.docx]

Table S1. Fluorescence emission maxima of HTL treated cyt-c, α-LA and lysoz

HTL conecentration (mM)	Fluorescence Emission maxima (nm)		
	cyt-c	α-Lactalbumin	Lysozy
0	332	332	349
1	356	351	349
2	357	351	349
